# Supplementary figures and images for: Di-arginine and FFAT-like motifs retain a subpopulation of PRA1 at ER-mitochondria membrane contact sites
Source: PLoS One. 2020 Dec 1;15(12):e0243075. doi: 10.1371/journal.pone.0243075 (PMC7707580; doi:10.1371/journal.pone.0243075)

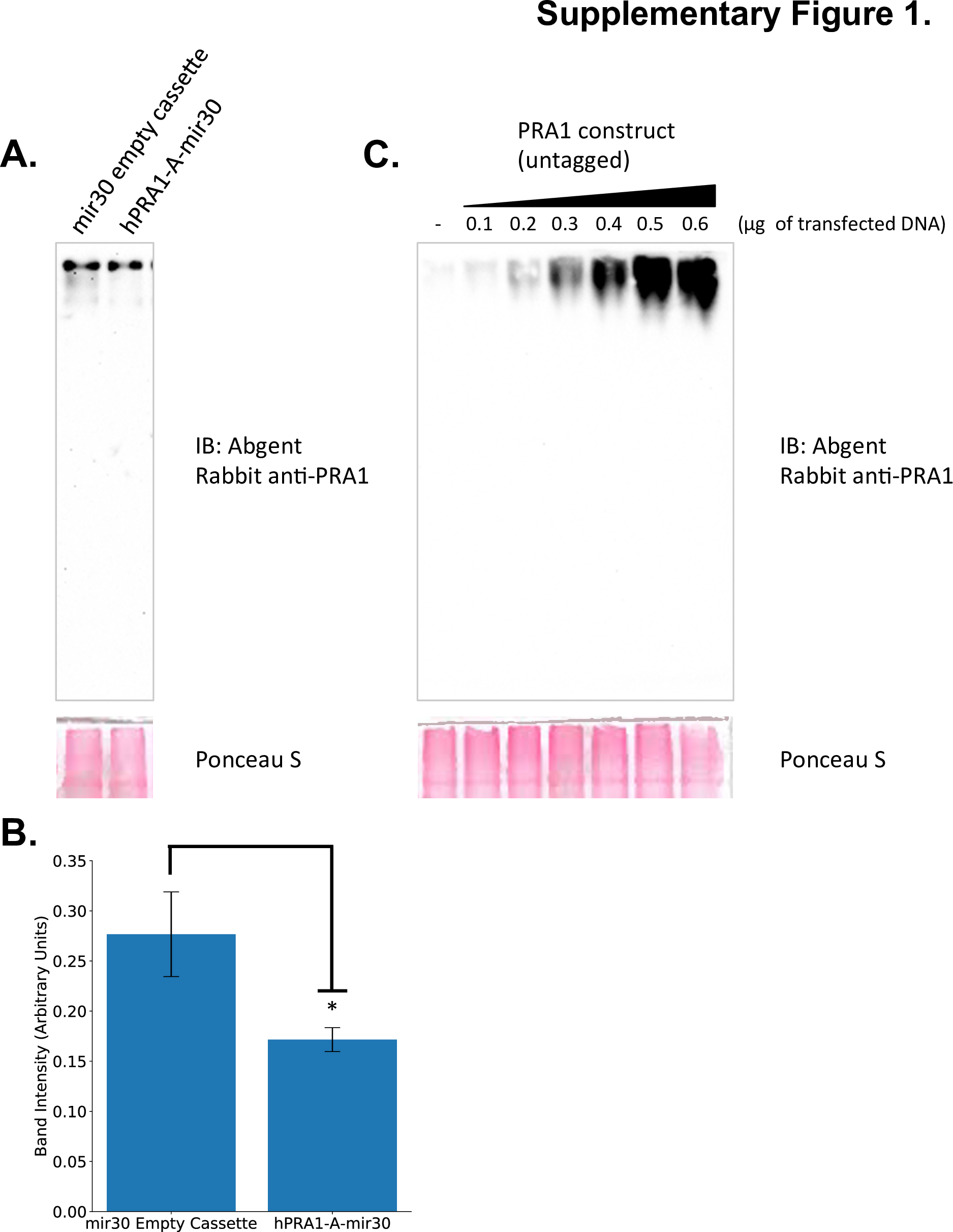

Supplement: S1 Fig — (A) Stably transfected polyclonal Hek293T cell lines that express both an empty and hPRA1 targeting mir30 cassettes were generated. PRA1 knock-down leads to a depletion of bands detected by the Abgent anti-PRA1 antibody. (B) Quantification of hPRA1 knockdown [n = 3, * p<0.05] (C) An increasing amount of an untagged mouse PRA1 cDNA construct was expressed in Hek293T cells. The bands detected using the Abgent anti-PRA1 antibody correspond in size and increase in intensity in accordance with the increasing amount of construct delivered to Hek293T cells. (TIF) [file pone.0243075.s001.tif]

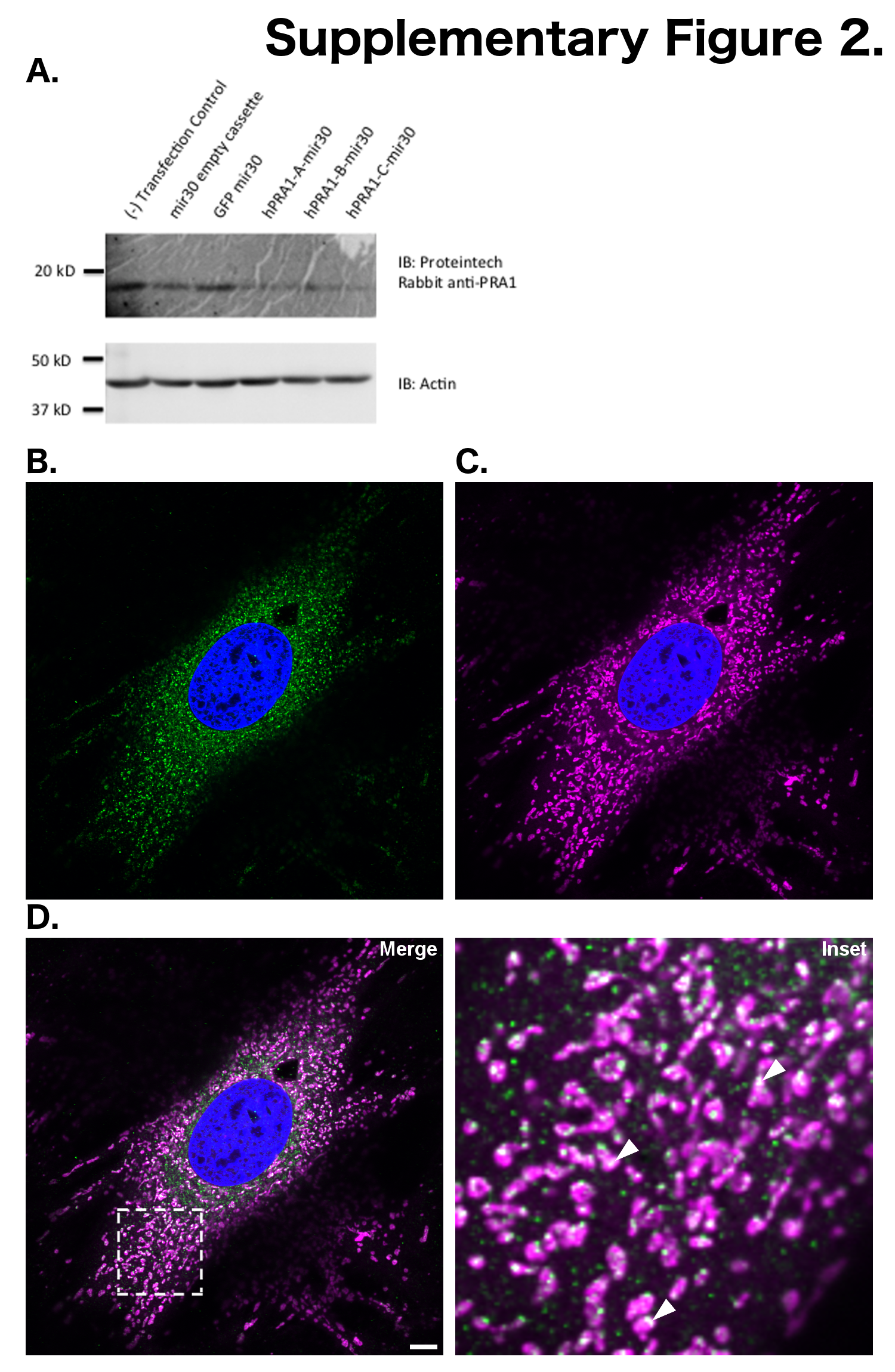

Supplement: S2 Fig — (A) A band is detected at the predicted size by the proteintech rabbit anti-PRA1 antibody (Cat. #10542-1-AP) after standard western analysis. The detected band is depleted upon targeted knock-down in Hek293T cells. (B) Immunohistochemical localization of PRA1 using the Proteintech anti-PRA1 antibody and the MitoTracker marker (C) in NIH3T3 cells shows co-localization (see D). Solid arrowheads denote regions of co-localization. Images are representative of at least three acquisitions and are compiled from a 1 μm z-section. The inset is a magnification of an area within the displayed images, highlighted with a dashed-line. Scale bar: 10 μm. Green: PRA1, Magenta: MitoTracker, Blue: Nuclear labeling via DAPI, White: Co-localization. (TIF) [file pone.0243075.s002.tif]

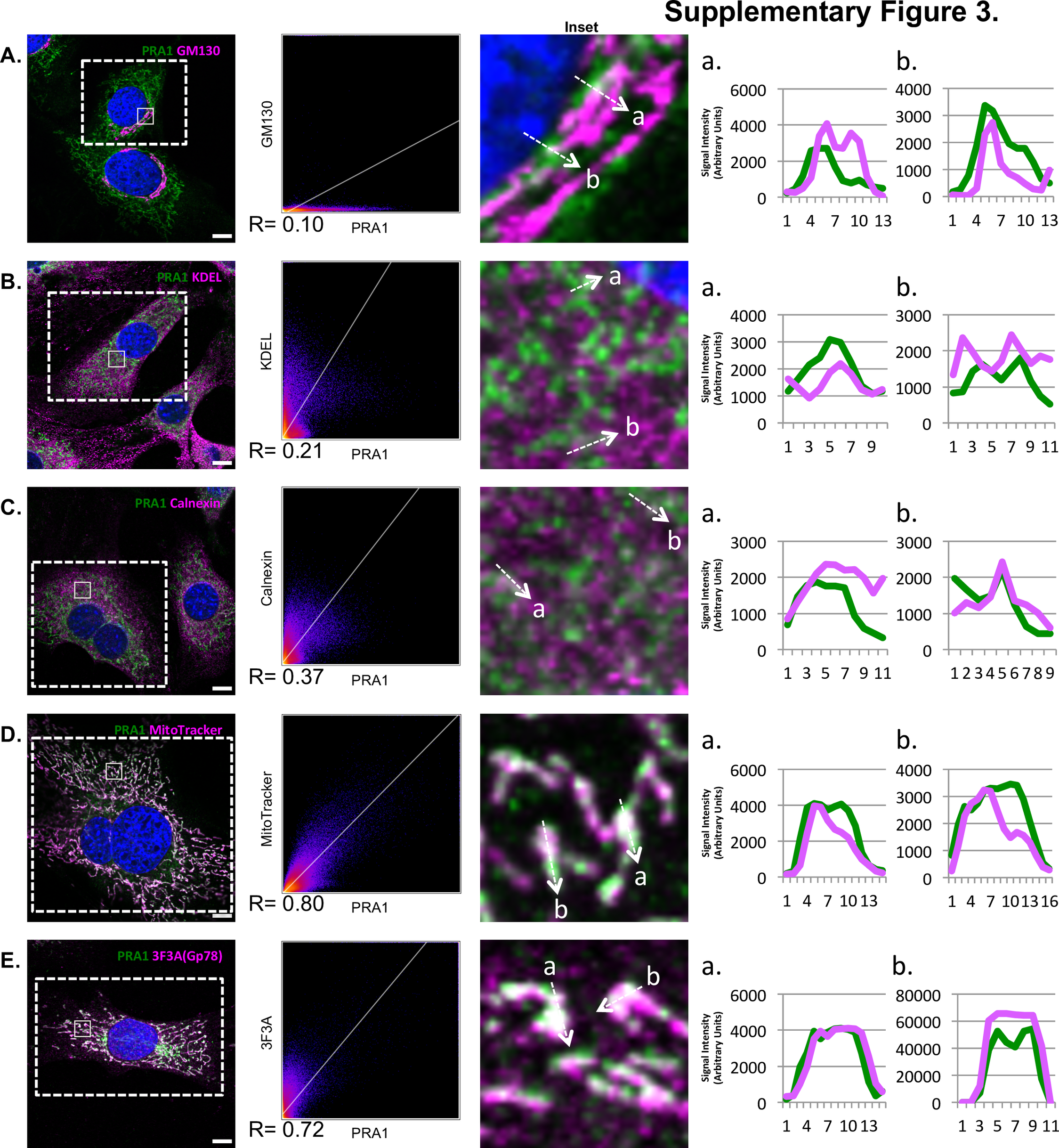

Supplement: S3 Fig — PRA1 was co-stained with (A) GM130, (B) KDEL, (C) Calnexin, (D) MitoTracker, and (E) Gp78/AMFR (3F3A antibody) as presented in Fig 2. Line-scans are annotated within each inset using a dashed-line in the direction of the arrow. Colocalization analysis and Pearson’s R value was acquired using the Coloc 2 plugin within ImageJ for a square region of interest encompassing a whole cell (dashed-line). Images are compiled from a 1 μm z-section. Insets are ten-fold magnifications of an area within the displayed images, highlighted with a solid-line. Scale bar: 10 μm. Green: PRA1, Magenta: Organelle specific marker, Blue: Nuclear labeling via DAPI, White: Co-localization. (TIF) [file pone.0243075.s003.tif]
