# Supplementary material for: Di-arginine and FFAT-like motifs retain a subpopulation of PRA1 at ER-mitochondria membrane contact sites
Source: PLoS One. 2020 Dec 1;15(12):e0243075. doi: 10.1371/journal.pone.0243075 (PMC7707580; doi:10.1371/journal.pone.0243075)
Supplement: S1 Table — (DOCX) [file pone.0243075.s004.docx]

**Table S1. Primer sequences used in this study**

| MluI_PRA1_1+FWD | GCTGCG**ACGCGT**GCGGCCCAGAAGGACCAGCAG |
| --- | --- |
| BglII_Stop_PRA1-185_RVRS | GCTGCG**AGATCT**TTACACAGGTTCCATCTGCAGCTCCTC |
| XhoI_PRA1_1+_FWD | GCTGCG**CTCGAG**ATGGCGGCCCAGAAGGACCAGCAG |
| MscI_PRA1_185+_RVRS | GATACAC**TGGCCA**CCACAGGTTCCATCTGCAGCTC |
| XbaI_XhoI_ATG_EGFP_FWD | GCTGCG**TCTAGA**CTCGAG**ATG**GTGAGCAAGGGCGAGGAG |
| BglII_STOP_MluI_EGFP_RVRS | GCTGCG**AGATCT**TCATCA**ACGCGT**CTTGTACAGCTCGTCCATGCC |
| mCHERRY_FWD_5’Cloning | GCTGCG**GAATTC**GACGCT**ACGCGT**GTGAGCAAGGGCGAGGAGGATAAC |
| mCHERRY_RVRS_5’Cloning | GCTGCG**AGATCT**TTA**CTTGTCATCGTCGTCCTTGTAGTC**CTTGTACAGCTCGTCCATGCCGCC |
| mCHERRY_FWD_3’Cloning | GCTGCG**GAATTC**CGCCACC**ATGGACTACAAGGACGACGATGACAAG**GTGAGCAAGGGCGAGGAGGATAAC |
| mCHERRY_RVRS_3’Cloning | GCTGCG**AGATCT**TTACGT**ACGCGT**CTTA**GGTACC**CTTGTACAGCTCGTCCATGCCGCC |
| KpnI_mSec61b_FWD | GCTGCG**GGTACC**CCGGGTCCAACGCCCAGTGGCACC |
| KpnI_mSec61b_RVRS | GCTGCG**ACGCGT**TTATGATCGCGTGTACTTGCCCCAAAT |
| XbaI_mMannII_FWD | GCTGCG**TCTAGA**ATGAAGTTAAGTCGCCAGTTCACCGTG |
| Mlu_mMannII-aa116_RVRS | GCTGCG**ACGCGT**CAAACAGTCTCTGGGGTCAGCCTG |
| KpnI_ PRA1_131+RVRS | GCTGCG**GGTACC**CTGATGTGCTGGGCTCACCTC |
| PRA1_R37/38/39/43A_FWD | **GAGGCCGCCGCCGCGACCATCGCCCCCTGG** |
| PRA1_R37/38/39/43A_RVRS | GGCGATGGTCGCGGCGGCGGCCTCCAG |
| PRA1_R37/38/39K_FWD | CTGGAGAAGAAGAAGGCGACCATCCGG |
| PRA1_R37/38/39K_RVRS | CCGGATGGTCGCCTTCTTCTTCTCCAG |
| PRA1_80-86_OE_FWD | TTCGTGTTTCTCGGCCTCATC |
| PRA1_72-78A_RVRS | **GCCGAGAAACACGAA**CACGGCGGCGGCGGCGGCGGCGGCCACGTT |
| mPRA1_66-86_72-78Δ_RVRS | GATGAGGCCGAGAAACACGAACACCACGTTGCGTACCAGGCG |
| mPRA1_66-86_72-79Δ_RVRS | GATGAGGCCGAGAAACACGAACACGTTGCGTACCAGGCG |
| mPRA1_YYQSNY-FFDAQE | GAGAAACACGAACAC**CTCCTGGGCGTCGAAGAA**CTCCAC |
